# Supplementary material for: Deep tissue space-gated microscopy via acousto-optic interaction
Source: Nat Commun. 2020 Feb 5;11:710. doi: 10.1038/s41467-020-14514-7 (PMC7002486; doi:10.1038/s41467-020-14514-7)
Supplement: Supplementary file 1 — Supplementary Information [file 41467_2020_14514_MOESM1_ESM.pdf]

Supplementary Information

Deep tissue space-gated microscopy via acousto-optic interaction

Jang *et al.*

Supplementary Note 1. Relative magnitudes of the contributing terms to the measured field

Without the space gating, the transmitted field $E(\mathbf{r}_\mathrm{d};\mathbf{r}_\mathrm{i})$ at the detection point $\mathbf{r}_\mathrm{d}$ for the illumination point $\mathbf{r}_\mathrm{i}$ is described as

$$E(\mathbf{r}_\mathrm{d};\mathbf{r}_\mathrm{i}) = \int_R T_\mathrm{i}(\mathbf{r}_\mathrm{o};\mathbf{r}_\mathrm{i})T_\mathrm{d}(\mathbf{r}_\mathrm{o};\mathbf{r}_\mathrm{d})d\mathbf{r}_\mathrm{o}$$

$$= \int_R [S(\mathbf{r}_\mathrm{o};\mathbf{r}_\mathrm{i})S(\mathbf{r}_\mathrm{o};\mathbf{r}_\mathrm{d})e^{-\frac{(L_\mathrm{i}+L_\mathrm{d})}{2l_\mathrm{s}}}$$

$$+S(\mathbf{r}_\mathrm{o};\mathbf{r}_\mathrm{i})e^{-\frac{L_\mathrm{i}}{2l_\mathrm{s}}}M_\mathrm{d}(\mathbf{r}_\mathrm{o};\mathbf{r}_\mathrm{d}) + S(\mathbf{r}_\mathrm{o};\mathbf{r}_\mathrm{d})e^{-\frac{L_\mathrm{d}}{2l_\mathrm{s}}}M_\mathrm{i}(\mathbf{r}_\mathrm{o};\mathbf{r}_\mathrm{i}) + M_\mathrm{i}(\mathbf{r}_\mathrm{o};\mathbf{r}_\mathrm{i})M_\mathrm{d}(\mathbf{r}_\mathrm{o};\mathbf{r}_\mathrm{d})]d\mathbf{r}_\mathrm{o} \quad (1)$$

Here, $\mathbf{r}_\mathrm{d}$ is set conjugate to $\mathbf{r}_\mathrm{i}$ for the confocal detection. The first term in the integral is the signal field $E_\mathrm{S}(\mathbf{r}_\mathrm{d};\mathbf{r}_\mathrm{i})$, and the other three terms contribute to the noise field $E_\mathrm{M}(\mathbf{r}_\mathrm{d};\mathbf{r}_\mathrm{i})$. In the following, we compare the relative magnitude between the four terms in the integral.

Let us compare the magnitude of the first term with those of the second and third terms. In fact, the magnitudes of the second and third terms should be smaller than that of the first term for the space-gated microscopy to work, i.e.

$$S(\mathbf{r}_\mathrm{o};\mathbf{r}_\mathrm{i})S(\mathbf{r}_\mathrm{o};\mathbf{r}_\mathrm{d})e^{-\frac{(L_\mathrm{i}+L_\mathrm{d})}{2l_\mathrm{s}}} > S(\mathbf{r}_\mathrm{o};\mathbf{r}_\mathrm{i})e^{-\frac{L_\mathrm{i}}{2l_\mathrm{s}}}M_\mathrm{d}(\mathbf{r}_\mathrm{o};\mathbf{r}_\mathrm{d}) \text{ or } S(\mathbf{r}_\mathrm{o};\mathbf{r}_\mathrm{d})e^{-\frac{L_\mathrm{d}}{2l_\mathrm{s}}}M_\mathrm{i}(\mathbf{r}_\mathrm{o};\mathbf{r}_\mathrm{i}). \quad (2)$$

Because only the fourth term $M_\mathrm{i}(\mathbf{r}_\mathrm{o};\mathbf{r}_\mathrm{i})M_\mathrm{d}(\mathbf{r}_\mathrm{o};\mathbf{r}_\mathrm{d})$ can be reduced with the space gating, Eq. (2) has to be met to ensure proper space-gated imaging. If the second or third term is larger than the first term, τ^{SG} would be unavoidably smaller than 1 even when $M_\mathrm{i}(\mathbf{r}_\mathrm{o};\mathbf{r}_\mathrm{i})M_\mathrm{d}(\mathbf{r}_\mathrm{o};\mathbf{r}_\mathrm{d})$ converges to 0 with the space gating. The condition Eq. (2) is satisfied when $|M_\mathrm{d}(\mathbf{r}_\mathrm{o};\mathbf{r}_\mathrm{d})| < \left|S(\mathbf{r}_\mathrm{o};\mathbf{r}_\mathrm{d})e^{-\frac{L_\mathrm{d}}{2l_\mathrm{s}}}\right|$ and $|M_\mathrm{i}(\mathbf{r}_\mathrm{o};\mathbf{r}_i)| < \left|S(\mathbf{r}_\mathrm{o};\mathbf{r}_\mathrm{i})e^{-\frac{L_\mathrm{i}}{2l_\mathrm{s}}}\right|$ at the confocal point on the object plane (i.e. the object point conjugate to $\mathbf{r}_\mathrm{i}$ or $\mathbf{r}_\mathrm{d}$). This condition is met when the ballistic components of the transfer functions ($T_\mathrm{i}(\mathbf{r}_\mathrm{o};\mathbf{r}_\mathrm{i}) = S(\mathbf{r}_\mathrm{o};\mathbf{r}_\mathrm{i})e^{-\frac{L_\mathrm{i}}{2l_\mathrm{s}}} + M_\mathrm{i}(\mathbf{r}_\mathrm{o};\mathbf{r}_\mathrm{i})$ and $T_\mathrm{d}(\mathbf{r}_\mathrm{o};\mathbf{r}_\mathrm{d}) = S(\mathbf{r}_\mathrm{o};\mathbf{r}_\mathrm{d})e^{-\frac{L_\mathrm{d}}{2l_\mathrm{s}}} + M_\mathrm{d}(\mathbf{r}_\mathrm{o};\mathbf{r}_\mathrm{d})$) appear as a

peak on the object plane. As shown in Fig. 1c, the ballistic component appeared as a peak in the typical transfer functions measured in our experiments. This also means that the confocal imaging can be successfully performed through either the illumination or detection part of the scattering medium. Therefore, these conditions are not stringent at all. For instance, let us consider imaging objects located at the center of a $20l_s$-thick scattering medium, i.e. $L_i + L_d = 20l_s$. Then, Eq. (2) is satisfied simply when it is possible to imaging through a $10l_s$-thick scattering medium, not $20l_s$-thick scattering medium. That is, ideally, space gating in effect reduces the thickness of the scattering medium to the half.

The other assumption we made in the derivation is that the magnitude of the fourth term is larger than those of the second and third terms:

$$\left|\int_{R\text{ or }R_{\mathrm{SG}}} M_{\mathrm{i}}(\mathbf{r}_{\mathrm{o}};\mathbf{r}_{\mathrm{i}})M_{\mathrm{d}}(\mathbf{r}_{\mathrm{o}};\mathbf{r}_{\mathrm{d}})\mathrm{d}\mathbf{r}_{\mathrm{o}}\right|$$
$$> \left|\int_{R\text{ or }R_{\mathrm{SG}}} S(\mathbf{r}_{\mathrm{o}};\mathbf{r}_{\mathrm{i}})e^{-\frac{L_{\mathrm{i}}}{2l_{\mathrm{s}}}}M_{\mathrm{d}}(\mathbf{r}_{\mathrm{o}};\mathbf{r}_{\mathrm{d}})\text{ or }S(\mathbf{r}_{\mathrm{o}};\mathbf{r}_{\mathrm{d}})e^{-\frac{L_{\mathrm{d}}}{2l_{\mathrm{s}}}}M_{\mathrm{i}}(\mathbf{r}_{\mathrm{o}};\mathbf{r}_{\mathrm{i}})\mathrm{d}\mathbf{r}_{\mathrm{o}}\right|. \quad (3)$$

This assumption guarantees that the fourth term is the dominant term in determining the noise field $E_{\mathrm{M}}(\mathbf{r}_{\mathrm{d}};\mathbf{r}_{\mathrm{i}})$ or $E_M^{\mathrm{SG}}(\mathbf{r}_{\mathrm{d}};\mathbf{r}_{\mathrm{i}})$. Equation (3) can be met when the ballistic wave is much weaker than the 'total' multiply scattered waves through either the illumination or detection part of the sample (i.e. $\left|\int_{R\text{ or }R_{\mathrm{SG}}} M_{\mathrm{i}}(\mathbf{r}_{\mathrm{o}};\mathbf{r}_{\mathrm{i}})\mathrm{d}\mathbf{r}_{\mathrm{o}}\right| > \left|\int_{R\text{ or }R_{\mathrm{SG}}} S(\mathbf{r}_{\mathrm{o}};\mathbf{r}_{\mathrm{d}})e^{-\frac{L_{\mathrm{d}}}{2l_{\mathrm{s}}}}\mathrm{d}\mathbf{r}_{\mathrm{o}}\right|$ or $\left|\int_{R\text{ or }R_{\mathrm{SG}}} M_{\mathrm{d}}(\mathbf{r}_{\mathrm{o}};\mathbf{r}_{\mathrm{d}})\mathrm{d}\mathbf{r}_{\mathrm{o}}\right| > \left|\int_{R\text{ or }R_{\mathrm{SG}}} S(\mathbf{r}_{\mathrm{o}};\mathbf{r}_{\mathrm{i}})\, e^{-\frac{L_{\mathrm{i}}}{2l_{\mathrm{s}}}}\mathrm{d}\mathbf{r}_{\mathrm{o}}\right|$). Considering that the multiply scattered wave extends to the wide area on the object plane and the ballistic wave decays exponentially with depth, the integral of the multiply scattered wave is typically larger than that of the ballistic component. This was still the case with the space gating when the integration range was reduced to R_{SG} for either the

illumination part or the detection part of the scattering medium. According to our analysis on the transfer functions shown in Supplementary Fig. 6, in our experimental configuration where space gating was as large as 30 µm × 70 µm and $\frac{L_\mathrm{i}}{l_\mathrm{s}}$ or $\frac{L_\mathrm{d}}{l_\mathrm{s}} > 10$, the ballistic transmission was consistently smaller than the scattered transmission within the spatial window space gating. Therefore, we could safely ignore the second and third terms in our theoretical description and estimate the noise suppression factor by considering the magnitude of the fourth term.

Finally, let us make a comparison between the first and fourth terms. Although the first term is initially smaller than the fourth term before the application of the space gating, it can be made larger than the fourth term with the space gating. This is due to the reduction of the fourth term by η times by the space gating, which is the main working principle of space gating to improve the imaging fidelity (i.e. $\tau_{\mathrm{SG}} = \eta \times \tau$) and to make diffraction-limited imaging possible (i.e. $\tau < 1$ and $\tau_{\mathrm{SG}} > 1$) .

Supplementary Note 2. Imaging depth versus the size of space gating and the optical wavelength

In the Principles section, it has been shown that the η is translated into logarithmic gain in the imaging depth $\Delta L = l_\mathrm{s} \times \log \eta$. The dependence of imaging depth on the size of acoustic focus and the optical wavelength can thus be deduced from η . As η is approximated as $\min\left(w_{\mathrm{M_i}}, w_{\mathrm{M_d}}\right)^2 / w_{\mathrm{SG}}^2$ the increase in the imaging depth is given by $2 l_\mathrm{s} \log[\min\left(w_{\mathrm{M_i}}, w_{\mathrm{M_d}}\right) / w_{\mathrm{SG}}]$. $w_{\mathrm{M_i}}$ and $w_{\mathrm{M_d}}$ are respectively the widths of the multiply scattered waves in the illumination and detection transfer functions, and w_{SG} is the width of the space gating set by the size of the acoustic

focus. Therefore, for the imaging depth increase of more than $\alpha \times l_\mathrm{s}$, w_SG needs to be smaller than $e^{-\alpha/2} \times \min(w_{\mathrm{M_i}}, w_{\mathrm{M_d}})$.

The optical wavelength affects to $w_{\mathrm{M_i}}$ and $w_{\mathrm{M_d}}$, which constitute the numerator of η. Because the anisotropy constant g and the absorption mean free path l_a are relatively slowly varying with respect to the wavelength, the major parameter affected is l_s. The use of longer wavelength increases l_s, which results in the proportionally larger imaging depth L because the intensity of the ballistic wave follows the Beer-Lambert law dictated by L/l_s. The effect of space gating η would then quadratically increase with L because $w_{\mathrm{M_i}}$ and $w_{\mathrm{M_d}}$ increase linearly with L in a diffusion regime. In a sub-diffusion regime, $w_{\mathrm{M_i}}$ and $w_{\mathrm{M_d}}$ increase at a higher rate with L. From the relation $\Delta L = l_\mathrm{s} \times \log \eta$, this translates into the additional gain in imaging depth. For example, if l_s becomes twice, $\Delta L/l_s$ is increased by $\log 4 \sim 1.4$ times.

The discussion to this point has focused on the increase of imaging depth by the space gating relative to that of the conventional imaging methods. To obtain the absolute imaging depth, one may need to estimate the imaging depth of conventional imaging methods. This requires the consideration of many parameters such as the optical parameters of scattering medium (l_s, l_a, and g), imaging configuration (e.g. the positions of scattering medium and imaging target), the numerical aperture and field-of-view of objective lens, and the effect of confocal and temporal gating. Such analysis has been of great interest in the field of biological imaging and could be found in the many previous studies[1,2].

Supplementary Note 3. Space gating and coherence gating in a forward scattering regime

In this work, we combined space gating with confocal gating to demonstrate the effectiveness of space gating in deep-tissue imaging. As the space gating is largely independent of the coherence

gating, its combination with the coherence gating can lead to additional gain in the imaging depth. However, in a forward scattering sample, the effect of coherence gating is coupled with the effect of space gating because the early-arriving waves that the coherence gating detects in the confocal geometry are spatially confined on the object plane. More specifically, in a forward scattering sample where the thickness of the medium is smaller than the transport mean free path, the propagating waves can be approximated as the collection of straight scattered rays with different deflection angles. Because the scattered ray with deflection angle θ has the time-delay of $L(1/\cos\theta - 1)/c$ ($\sim L\theta^2/2$ for a small θ) where c is the average speed of light in the medium, the early-arriving waves that coherence gating (i.e. time gating) detects has a small θ while the later-arriving waves has a relatively larger θ. On the other hand, the scattered ray has the spatial spread of $2 \times L/2 \times \tan\theta$ ($\sim L\theta$ for a small θ) on the transverse plane of the object. Therefore, the arrival time and the spatial spread of forward-scattering waves are coupled in such a way that the early-arriving waves has a small circular spatial spread on the object plane while the later-arriving waves has a larger ring-shape spatial spread on the object plane.

When the coherence gating of $c \times t_{\mathrm{TG}}$ is set, the deflection angle θ is approximated to $\sqrt{2c \times t_{\mathrm{TG}}/L}$ and the corresponding spatial spread of the early-arriving waves on the object plane is given as $\sqrt{2L} \times \sqrt{c \times t_{\mathrm{TG}}}$. In other words, the coherence gating of $c\Delta t_{\mathrm{TG}}$ is equivalent to the space gating with the window size of $\sqrt{2L} \times \sqrt{c \times t_{\mathrm{TG}}}$. Assuming that the gating windows for coherence gating ($\mathrm{c} \times t_{\mathrm{TG}}$) and space gating ($w_{\mathrm{SG}}$) are of comparable size ($w \sim w_{\mathrm{SG}} \sim \mathrm{c} \times t_{\mathrm{TG}}$; typically tens of micrometers), the noise suppression factor of space gating is $2L/w$ ($= \left(\sqrt{2L} \times \sqrt{w}/w\right)^2$) times larger than that of coherence gating, due to the quadratic dependence of η to w. With L of 1 mm and w of 30 μm, the space gating can outperform coherence gating by

more than 30 times. This effectiveness of space gating in a forward scattering regime has been critical for our realization of phase imaging within a thick scattering medium, which has been challenging for the conventional imaging methods relied on coherence gating. In the regime where the thickness of the scattering medium sufficiently exceeds a transport mean free path, the coupling between coherence gating and space gating fades out, and their combined use will complement each other in such a way to jointly enhance noise suppression factor.

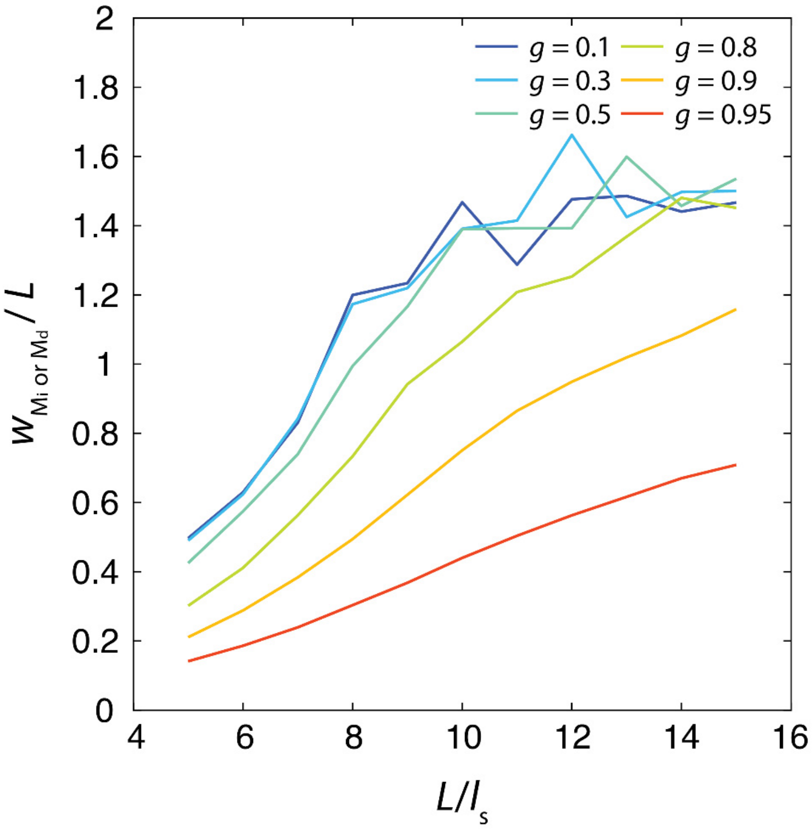

Supplementary Figure 1. Spatial extent of multiply scattered wave.

The spatial extent ($w_{\mathrm{M_i\ or\ M_d}}$) of the multiply scattered component of illumination (or detection) transfer functions $|M_{\mathrm{i}}(\mathbf{r}_{\mathrm{o}};\mathbf{r}_{\mathrm{i}})|^2$ (or $|M_{\mathrm{d}}(\mathbf{r}_{\mathrm{o}};\mathbf{r}_{\mathrm{d}})|^2$) is plotted over the optical thickness ($=L/l_{\mathrm{s}}$) range from 5 to 15 with different values of anisotropy coefficient g. $w_{\mathrm{M_i\ or\ M_d}}$ is normalized by the physical thickness of the medium L. $w_{\mathrm{M}_i\ \mathrm{or\ M_d}}$ is calculated using Monte Carlo simulation of light propagation through a scattering medium where the pencil beam is normally incident on a scattering medium and the position of exiting photons are sampled at the output side with the full acceptance angle of 90°. $|M_{\mathrm{i}}(\mathbf{r}_{\mathrm{o}};\mathbf{r}_{\mathrm{i}})|^2$ is approximated as a top-hat function $|M_{\mathrm{i}}(\mathbf{r}_{\mathrm{o}}=\mathbf{r}_{\mathrm{i}};\mathbf{r}_{\mathrm{i}})|^2\Pi(\mathbf{r}_{\mathrm{o}};\mathbf{r}_{\mathrm{i}})$, where $\Pi(\mathbf{r}_{\mathrm{o}};\mathbf{r}_{i})$ is 1 for the domain of $|\mathbf{r}_{\mathrm{o}}-\boldsymbol{r}_{\mathrm{i}}| < w_{\mathrm{M_i}}$ and 0 outside the domain. In a diffusive regime where $(1-g)L/l_{\mathrm{s}} \gg 1$, $\Delta w_{\mathrm{M_i\ or\ M_d}}$ is saturated to about $1.5L$ due to the

isotropic diffusion process. In contrast, for a highly forward-scattering medium (e.g. $g > 0.9$), $w_{\mathrm{M_i\ or\ M_d}}$ is significantly smaller than the saturated width even at L/l_s of 10. As explained in Principles, the effect of space gating can be approximated as $\eta = \min\left(w_{\mathrm{M_i}}, w_{\mathrm{M_d}}\right)^2 / w_{\mathrm{SG}}^2$ where w_{SG} is the width of space gating. Therefore, this plot can be used to roughly estimate the noise suppression effect of space gating. For biological tissues, $w_{\mathrm{M_i}}$ and $w_{\mathrm{M_d}}$ can be typically in the range from hundreds of microns to millimeters when $L/l_\mathrm{s}{\sim}10$, from which we can expect $\eta > 10$ if the size of space gating w_{SG} is as small as tens of microns, as is the case with a high-frequency acoustic focus. For instance, for a 500 μm-thick tissue slab with l_s = 50 μm and g = 0.9, $w_{\mathrm{M_i\ or\ M_d}}$ is about 380 μm from the yellow curve of $w_{\mathrm{M_i\ or\ M_d}}/L{\sim}0.75$. From this, we can expect the noise suppression effect η of 160 with a 30 μm-width space gating.

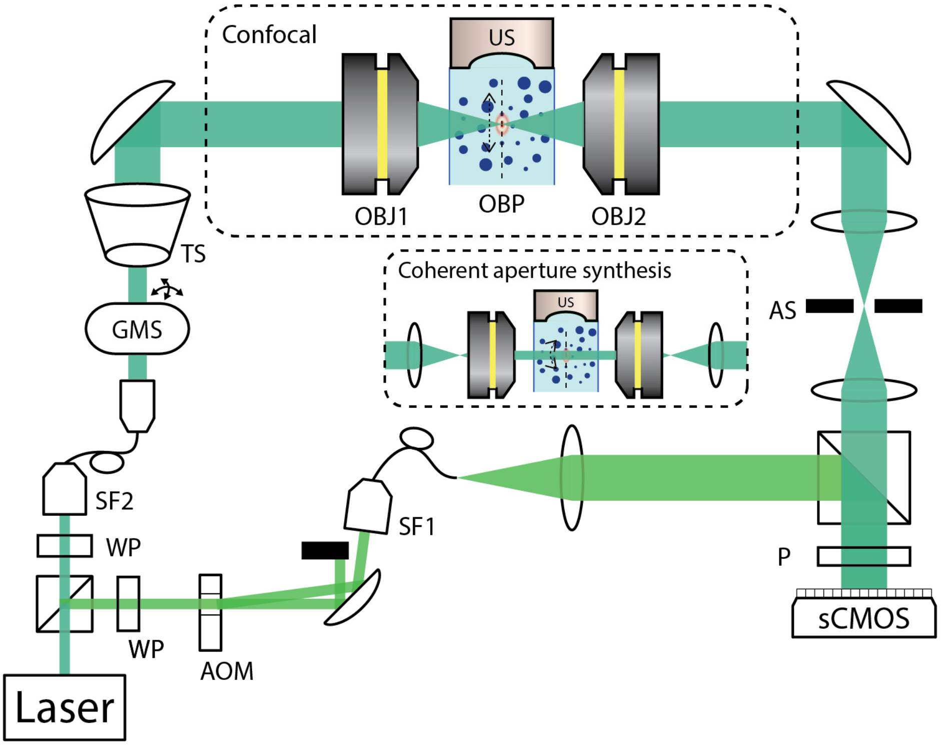

Supplementary Figure 2. Experimental setup.

Laser beam with 7-ns pulse width and 40 kHz repetition rate is split into signal and reference beams for phase-shifting interferometry. Both beams are spatially filtered through single-mode optical fibers. The collimated signal beam is relayed through a pair of galvanometer mirrors (GMS) and a telescope system (TS) to extend the beam waist. Signal beam is then either focused or collimated through an objective lens to illuminate the object. Corresponding beam paths are respectively indicated in the dashed boxes labeled as ‘Confocal’ and ‘Coherent aperture synthesis’. GMS is used to scan the position of focused spot or the angle of collimated beam incident on the object. Each laser pulse is synchronized with the 3 cycles of focused acoustic wave so that a portion of the signal beam is frequency-modulated through the acoustic focus formed at the object plane. The transmitted beam is collected by an objective lens and relayed to sCMOS camera so that the

object plane is conjugate to the detector plane. The reference beam is frequency-modulated through an acousto-optic modulator. The 1st order diffracted beam is spatially-filtered, collimated, and guided to the sCMOS camera for phase-shifting interferometry. OBP, object plane; OBJ, objective lens; WP, half-wave plate; SF, spatial-filtering system; AOM, acousto-optic modulator; US, ultrasound transducer; AS, aperture stop; P, polarizer.

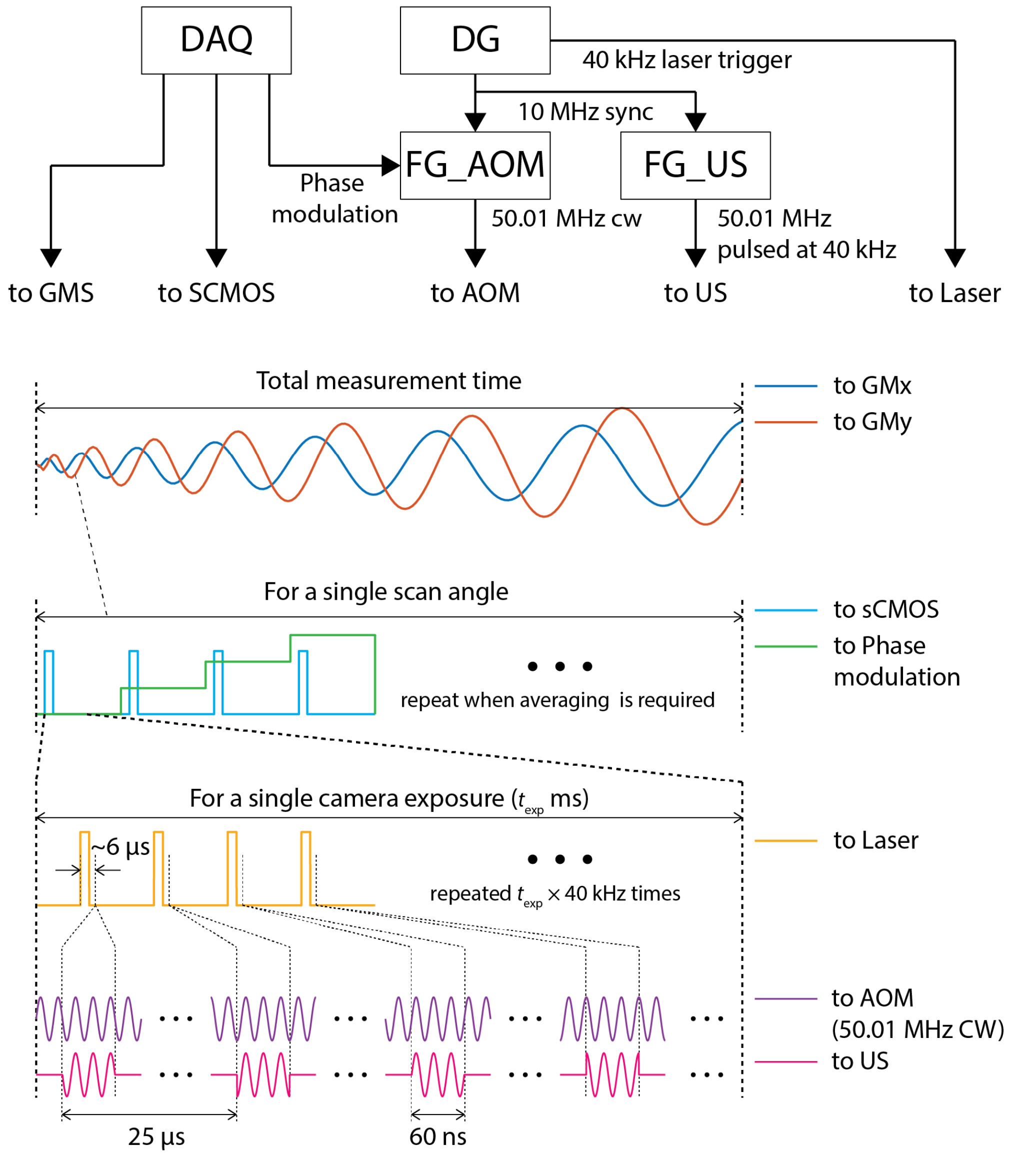

Supplementary Figure 3. Electrical signal flow.

A data acquisition (DAQ) board outputs two electrical signals to scan the angles of galvanometric mirrors (GMx and GMy), which performs rater scanning for confocal imaging or helical angular scanning for coherent aperture synthesis. For each scan angle, DAQ generates a stepping signal to modulate the phase of the 50.01-MHz signal driving an acousto-optic modulator (AOM). For four-step phase-shifting interferometry, the phase modulation signal is stepped through 4 values.

sCMOS camera is triggered at each phase step using the same DAQ. For each exposure period of the camera, hundreds of laser pulses are collected, which are triggered with the 40-kHz trigger signal from a digital delay generator. The same digital delay generator outputs a 10-MHz reference signal to synchronize the two function generators that controls an acousto-optic modulator and ultrasound transducer. The function generator for AOM outputs a 50.01-MHz sinusoidal signal whose phase is stepped by the signal from DAQ, and the function generator for ultrasound transducer outputs a three-cycles of 50.01-MHz sinusoidal signal with the repetition rate of 40 kHz. The initial phase of the three-cycle sine wave is repeatedly stepped through 0, $\pi/2$, π, and $3\pi/2$ to be coherent with the 50.01 MHz continuous sine wave. In other words, the relative phase between the 50.01 MHz continuous sine wave and the three-cycle sine wave was fixed within each exposure period to make the modulated wave from the transducer generate a static interference pattern with the reference wave. We precisely control the delay of the signal to the transducer in relation to the laser trigger signal to match the timing between the laser pulse and the acoustic pulse. Because the delay between the laser trigger signal and the actual pulse output was around 10 μs and the travel time of the acoustic wave was about 4 μs, the delay between the short-pulsed acoustic signal and the laser trigger signal was set to be ~ 6 μs.

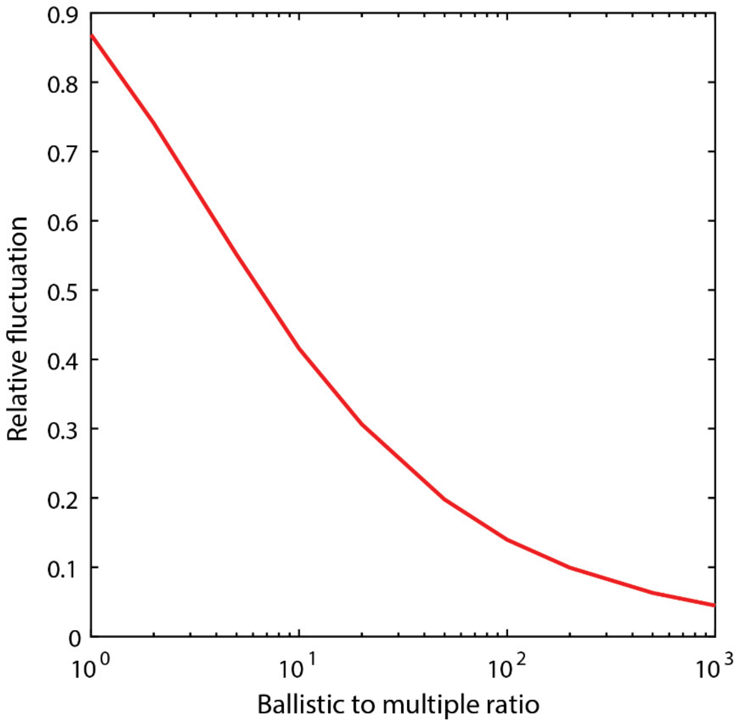

Supplementary Figure 4. Interference between ballistic and multiply scattered waves.

The relative fluctuation in the intensity recording $|E(\mathbf{r}_\mathrm{d};\mathbf{r}_\mathrm{i})|^2 = |E_\mathrm{S}(\mathbf{r}_\mathrm{d};\mathbf{r}_\mathrm{i}) + E_\mathrm{M}(\mathbf{r}_\mathrm{d};\mathbf{r}_\mathrm{i})|^2$ through a confocal pinhole is numerically calculated over a wide range of ballistic to multiply-scattered ratio $|E_\mathrm{S}(\mathbf{r}_\mathrm{d};\mathbf{r}_\mathrm{i})|^2/|E_\mathrm{M}(\mathbf{r}_\mathrm{d};\mathbf{r}_\mathrm{i})|^2$. The fluctuating phase between $E_\mathrm{S}(\mathbf{r}_\mathrm{d};\mathbf{r}_\mathrm{i})$ and $E_\mathrm{M}(\mathbf{r}_\mathrm{d};\mathbf{r}_\mathrm{i})$ causes the statistical fluctuations in $|E(\mathbf{r}_\mathrm{d};\mathbf{r}_\mathrm{i})|^2$. For the numerical calculation, the amplitude and phase of $E_\mathrm{M}(\mathbf{r}_\mathrm{d};\mathbf{r}_\mathrm{i})$ are randomly generated respectively based on the Rayleigh distribution and the uniform distribution within the range of 0 to 2π. $|E_\mathrm{S}(\mathbf{r}_\mathrm{d};\mathbf{r}_\mathrm{i})|^2$ is set to 1 and the average intensity of multiply scattered waves varies from 1 to 1,000. We performed 10,000 trials, and the relative fluctuation is defined by the ratio of the standard deviation of $|E(\mathbf{r}_\mathrm{d};\mathbf{r}_\mathrm{i})|^2$ to the average of $|E(\mathbf{r}_\mathrm{d};\mathbf{r}_\mathrm{i})|^2$. Even when the intensity of ballistic wave is 10 times larger than the average intensity of multiply scattered wave, the relative fluctuation is as large as 40 %. Due to this competing

nature between the ballistic and multiply scattered waves, the imaging contrast of conventional microscope is quickly degraded in a scattering medium. It also quantitatively explains how the noise suppression effect of gating operation improves the imaging contrast.

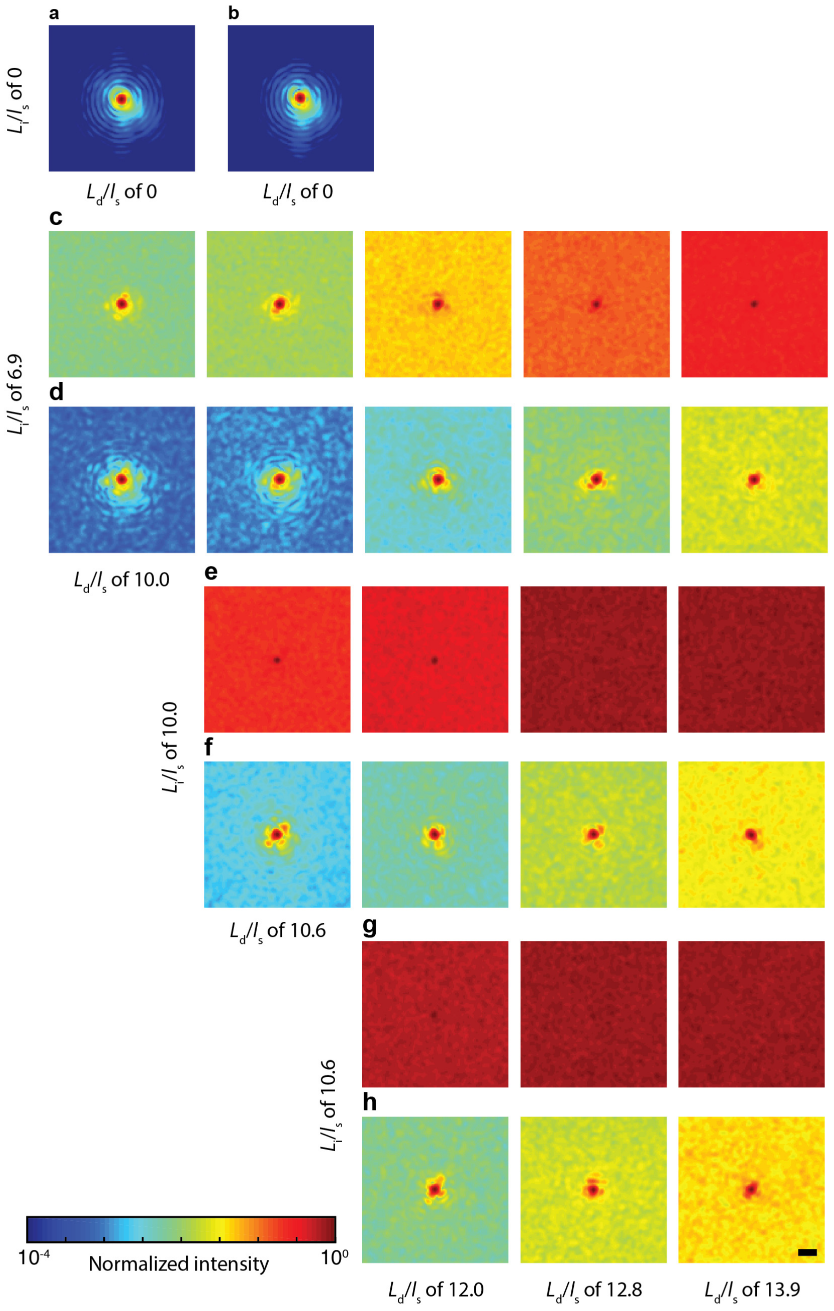

Supplementary Figure 5. Ensemble-averaged point spread functions (PSFs).

The PSFs without and with space-gating (i.e. $|E(\mathbf{r}_\mathrm{d};\mathbf{r}_\mathrm{i})|^2$ and $\left|E^{\mathrm{SG}}(\mathbf{r}_\mathrm{d};\mathbf{r}_\mathrm{i})\right|^2$) for different combinations of input and output scattering layers are measured at the detector plane for a specific illumination point r_i and ensemble-averaged for 50 different realizations of the scattering medium.

Illumination point is conjugate to the position of the peak at the center. **a** and **b,** The measured PSFs for a transparent medium without and with space gating, respectively. **c-h,** The measured PSFs, without space gating and width space gating, for a scattering medium whose total optical thickness $L_{\mathrm{tot}}/l_{\mathrm{s}}$ ranges from 16.9 to 24.5. $L_{\mathrm{i}}/l_{\mathrm{s}}$ and $L_{\mathrm{d}}/l_{\mathrm{s}}$ for each figure is indicated on the left and bottom sides of the figure, respectively. With the increase of optical thickness, the ballistic wave becomes obscured by the multiply scattered wave. **c, e,** and **g,** The measured PSFs, without space gating. **d ,f,** and **h,** The measured PSFs, with space gating. The configurations of the input and output layers are the same as in **c, e,** and **g**. The ballistic wave appeared as a peak with a contrast of 18 even at $L_{\mathrm{tot}}/l_{\mathrm{s}}$ of 24.5. The intensity maps are normalized by their maximum intensities. Scale bar: 5 µm.

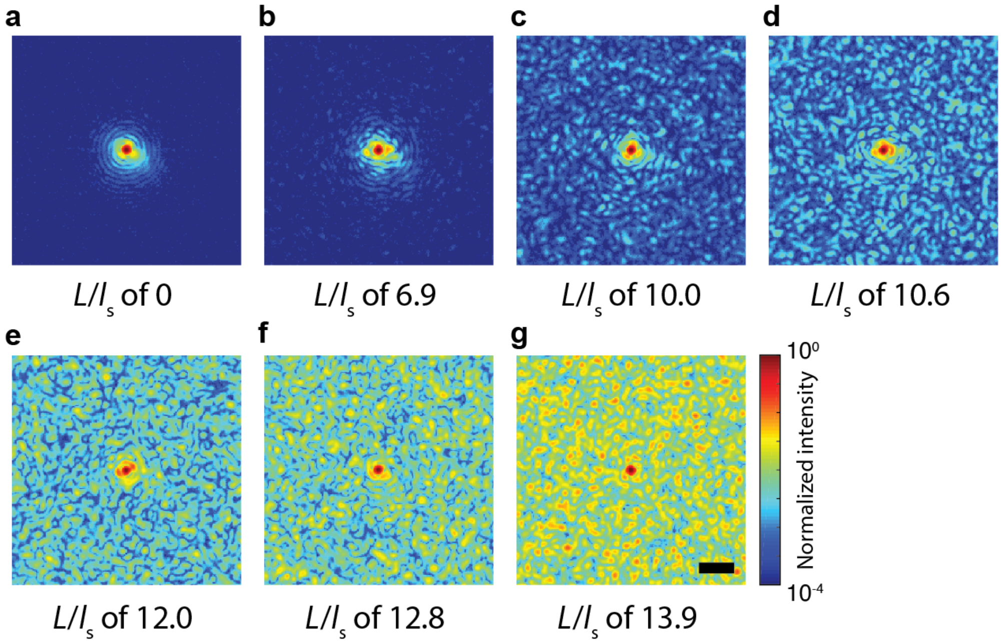

Supplementary Figure 6. Illumination and detection transfer functions.

a-g, Intensity maps of measured transfer functions on the object plane for the illumination or detection point located at the center (i.e. $|T_{\mathrm{i}}(\mathbf{r}_{\mathrm{o}};\mathbf{r}_{\mathrm{i}})|^2$ or $|T_{\mathrm{d}}(\mathbf{r}_{\mathrm{o}};\mathbf{r}_{\mathrm{d}})|^2$). The optical thickness of the input or output scattering layers L/l_{s} ranges from 0 to 13.9. With the increase of optical thickness, the relative intensity of the randomly fluctuating background speckle pattern becomes stronger and the spatial extent of multiply scattered wave is increased. Within the field of view in the presented figure, the increase in the spatial extent is only observable when the optical thickness is increased from 6.9 to 10. This increase explains the trend observed in Fig. 4b, where the noise suppression factor η is increased from 4~11 to 47~150 when $L_{\mathrm{i}}/l_{\mathrm{s}}$ is increased from 6.9 to 10 or 10.6. The intensity maps are normalized by their maximum intensities. Scale bar: 10 μm. See methods for the detailed measurement procedures for transfer functions.

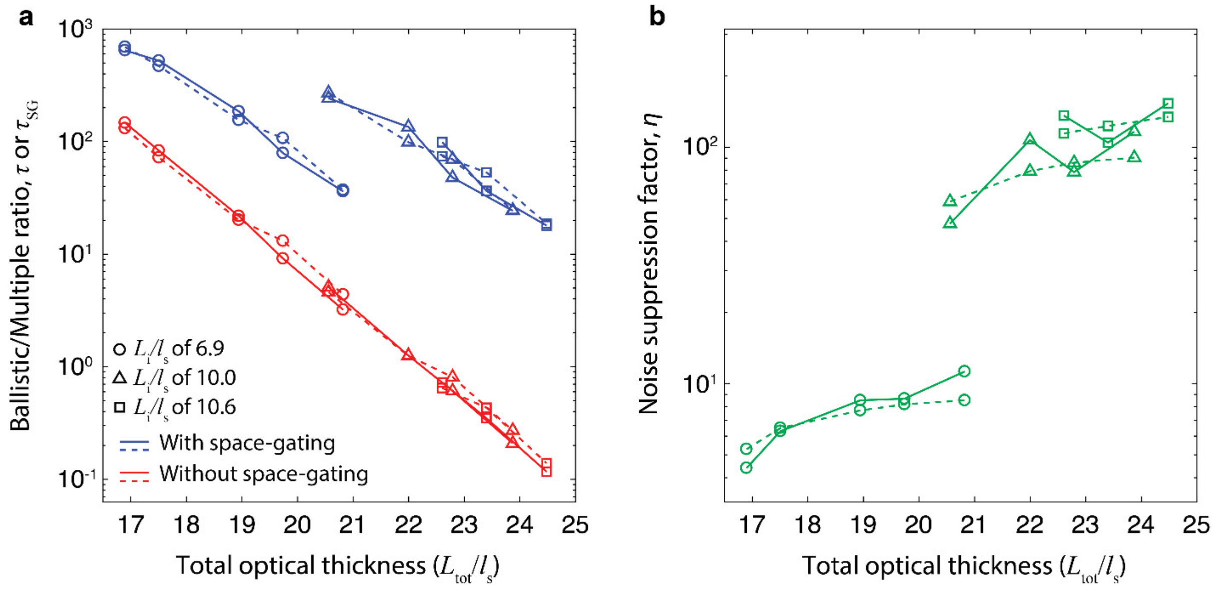

Supplementary Figure 7. Theoretical noise suppression efficiency of space gating.

a, Ratio of the ballistic to multiply scattered waves with (τ_{SG}, blue) and without space gating (τ, red) as a function of L_{tot}/l_s. Solid lines are directly measured from the PSFs in Supplementary Fig. 5, and dotted lines are estimated from the transfer functions in Supplementary Fig. 6 based on the model in Principle. Similar to Fig. 4, circular, triangular and rectangular markers indicate the cases when the optical thicknesses of the input layer, L_i/l_s, are fixed to 6.9, 10.0, and 10.6, respectively. The optical thickness of the output layer is varied for each case. The prediction from transfer functions shows a remarkable agreement with the ratios deduced from the direct measurement of PSFs. **b,** Noise suppression factor η of space gating. η is obtained from τ_{SG}/τ in **a**. The effect of space gating can be precisely estimated from the transfer functions.

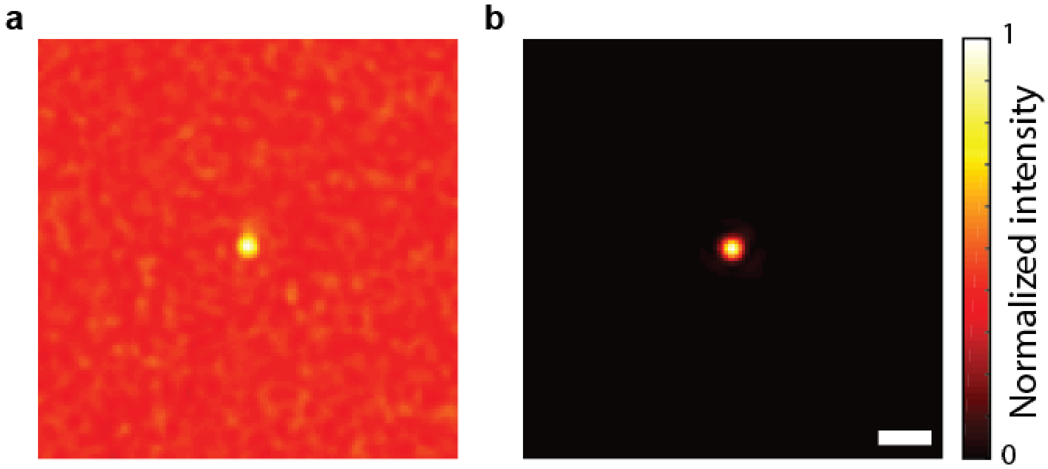

Supplementary Figure 8. Point spread functions through a tissue phantom.

The PSFs without and with space-gating (i.e. $|E(\mathbf{r}_\mathrm{d};\mathbf{r}_\mathrm{i})|^2$ and $\left|E^{\mathrm{SG}}(\mathbf{r}_\mathrm{d};\mathbf{r}_\mathrm{i})\right|^2$) are measured at the detector plane for a specific illumination point $\mathbf{r}_\mathrm{i}$ through a tissue phantom composed of 6-mm-thick PAA gel containing 0.8% of fat emersion. The PSFs are ensemble-averaged for 50 different realizations of the scattering medium. **a,** Without space gating, τ is measured to be 1.1. The focused ballistic wave is initially not visible in each PSF measured at the specific realization of scattering medium because of its interference with the multiply scattered wave (i.e. the peak intensity fluctuates by ~90 % as predicted from Supplementary Fig. 4). However, the spot becomes distinctly visible after the ensemble average, from which we can accurately quantify τ. **b,** With space gating, τ_{SG} is improved to 240. The intensity maps are normalized by their maximum intensities. Scale bar: 5 µm.

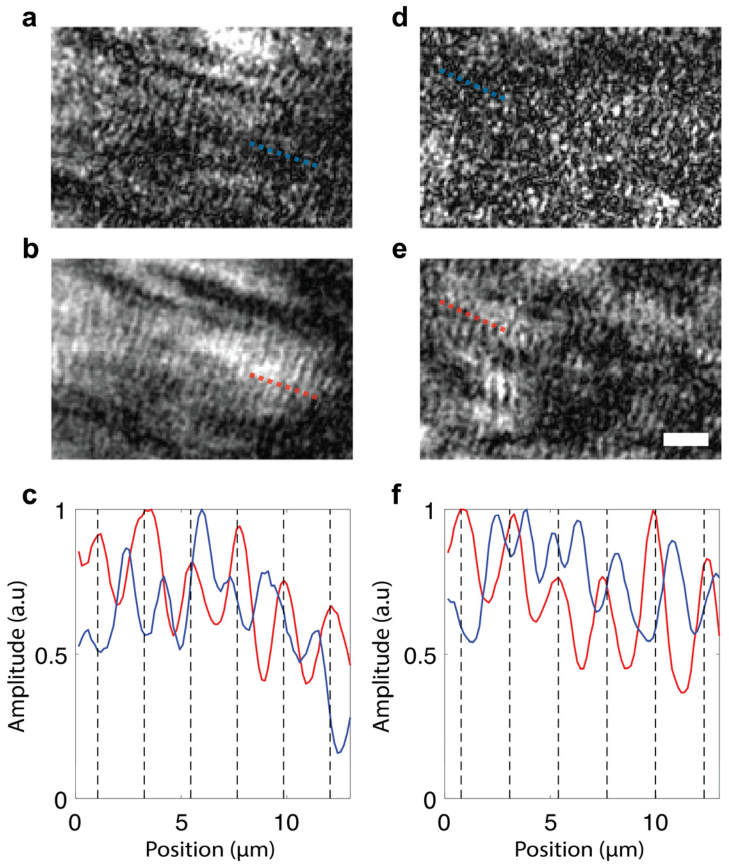

Supplementary Figure 9. Amplitude profiles along a muscle fiber in zebrafish.

a and **b,** Conventional and space-gated images from Figs. 6e and 6h, respectively. **c**, Amplitude profile along the dotted blue and red lines in **a** and **b**. With space gating, the alternating light and dark bands (i.e. a repeating unit of muscle fiber) appear periodically. **d** and **e,** Conventional and space-gated images from Figs. 6f and 6i, respectively. **f**, Amplitude profile along the dotted blue and red lines in **d** and **e**. Similar to **c**, the alternating light and dark bands appear periodically only with space gating. Scale bar: 10 µm.

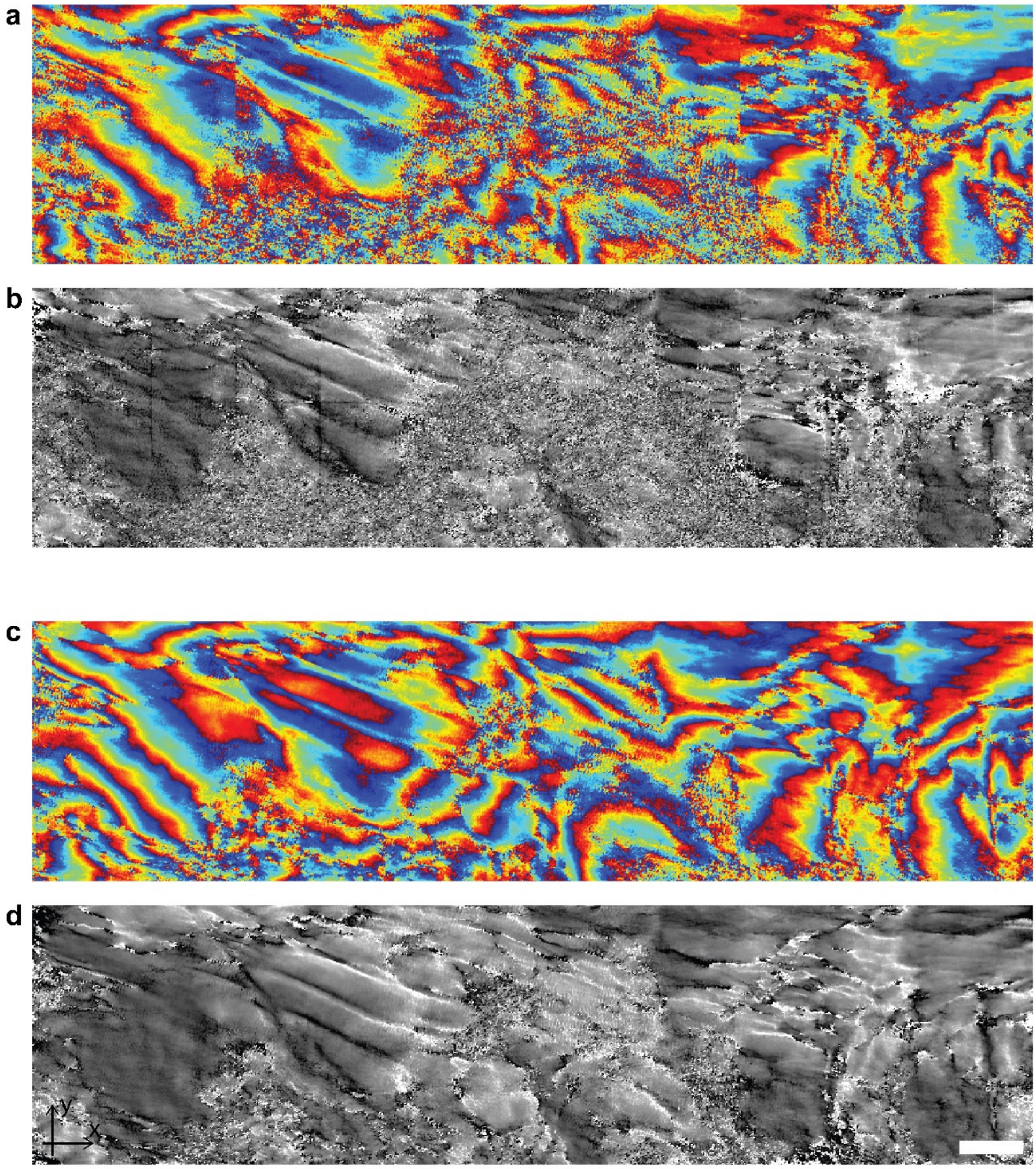

Supplementary Figure 10. Phase and phase-gradient images of a 30-dpf zebrafish.

The skeletal muscle structure of the head-trunk junction was imaged. **a** and **b,** Phase map and phase-gradient image without space gating. Both images present the speckle-like noise due to the effect of multiple light scattering. The structural features of myosepta and muscle-bone junction

are not clearly identified. **c** and **d,** Phase map and phase-gradient image with space gating. Space gating significantly suppresses the effect of multiple scattering and makes the structural features of myosepta and muscle-bone junction visible throughout the entire field-of-view. Phase-gradient image provides a clearer view of individual muscle fibers, even compared to the space-gated amplitude image in Fig. 6d. Scale bar: 50 μm.

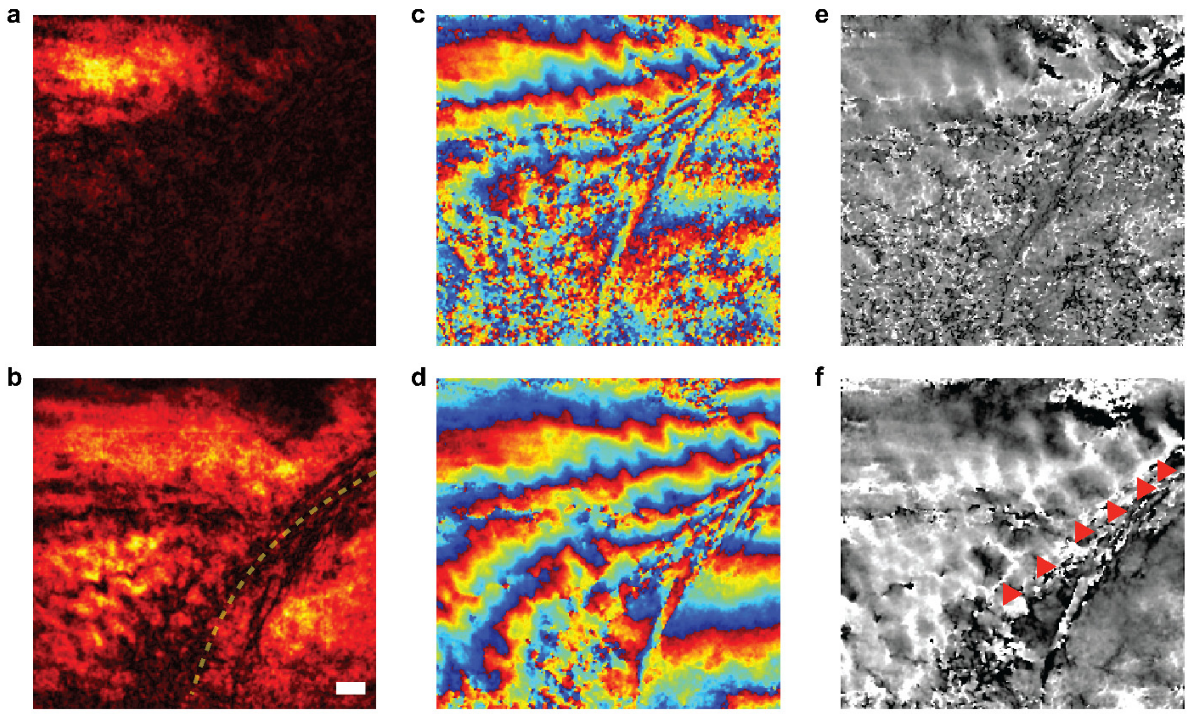

Supplementary Figure 11. Imaging of the cartilage structures within a 30-dpf zebrafish.

The cartilage structures are located around the junction of the otic and occipital bones. **a** and **b**, Amplitude images without and with space gating. The junction between the cartilages (dotted yellow line) is only visible with space gating. **c** and **d**, Phase maps without and with space gating. Speckle-like noise is significantly suppressed with space gating. **e** and **f,** Phase-gradient images without and with space gating. Space-gated phase-gradient image reveals the positions of the lacunae (i.e. cavities) within a cartilage (indicated with the red arrowheads). Scale bar: 10 μm.

Supplementary References

1. Hee, M. R., Swanson, E. A., Izatt, J. A., Jacobson, J. M. & Fujimoto, J. G. Femtosecond transillumination optical coherence tomography. *Opt. Lett.* **18**, 950–952 (1993).

2. Badon, A., Boccara, A. C., Lerosey, G., Fink, M. & Aubry, A. Multiple scattering limit in optical microscopy. *Opt. Express* **25**, 28914–28934 (2017).
