# Supplementary material for: Deep tissue space-gated microscopy via acousto-optic interaction
Source: Nat Commun. 2020 Feb 5;11:710. doi: 10.1038/s41467-020-14514-7 (PMC7002486; doi:10.1038/s41467-020-14514-7)
Supplement: Supplementary file 3 — Reporting Summary [file 41467_2020_14514_MOESM3_ESM.pdf]

nature research

Corresponding author(s): Mooseok Jang, Wonshik Choi

Last updated by author(s): Dec 27, 2019

Reporting Summary

Nature Research wishes to improve the reproducibility of the work that we publish. This form provides structure for consistency and transparency in reporting. For further information on Nature Research policies, see Authors & Referees and the Editorial Policy Checklist .

Statistics

For all statistical analyses, confirm that the following items are present in the figure legend, table legend, main text, or Methods section.

n/a	Confirmed	
x		The exact sample size (*n*) for each experimental group/condition, given as a discrete number and unit of measurement
	x	A statement on whether measurements were taken from distinct samples or whether the same sample was measured repeatedly
x		The statistical test(s) used AND whether they are one- or two-sided<br>*Only common tests should be described solely by name; describe more complex techniques in the Methods section.*
x		A description of all covariates tested
x		A description of any assumptions or corrections, such as tests of normality and adjustment for multiple comparisons
x		A full description of the statistical parameters including central tendency (e.g. means) or other basic estimates (e.g. regression coefficient) AND variation (e.g. standard deviation) or associated estimates of uncertainty (e.g. confidence intervals)
x		For null hypothesis testing, the test statistic (e.g. *F*, *t*, *r*) with confidence intervals, effect sizes, degrees of freedom and *P* value noted<br>*Give P values as exact values whenever suitable.*
x		For Bayesian analysis, information on the choice of priors and Markov chain Monte Carlo settings
x		For hierarchical and complex designs, identification of the appropriate level for tests and full reporting of outcomes
x		Estimates of effect sizes (e.g. Cohen's *d*, Pearson's *r*), indicating how they were calculated

Our web collection on statistics for biologists contains articles on many of the points above.

Software and code

Policy information about availability of computer code

Data collection	Custum Matlab codes were used to control the instruments including sCMOS camera, galvanometer mirrors, and micromanipulators. The The MATLAB codes are not central to the research. The MATLAB codes used in this work are available from the corresponding authors upon request.
Data analysis	Custum Matlab codes were used to convert the acquired datasets into the figures . As we used some common command lines in MATLAB, the custom MATLAB codes are not deemed central to the research. The MATLAB codes used in this work are available from the corresponding authors upon request.

For manuscripts utilizing custom algorithms or software that are central to the research but not yet described in published literature, software must be made available to editors/reviewers. We strongly encourage code deposition in a community repository (e.g. GitHub). See the Nature Research guidelines for submitting code & software for further information.

Data

Policy information about availability of data

All manuscripts must include a data availability statement. This statement should provide the following information, where applicable:

- Accession codes, unique identifiers, or web links for publicly available datasets
- A list of figures that have associated raw data
- A description of any restrictions on data availability

The datasets acquired for this study are available from the corresponding authors upon request.

Field-specific reporting

Please select the one below that is the best fit for your research. If you are not sure, read the appropriate sections before making your selection.

☒ Life sciences ☐ Behavioural & social sciences ☐ Ecological, evolutionary & environmental sciences

For a reference copy of the document with all sections, see nature.com/documents/nr-reporting-summary-flat.pdf

Life sciences study design

All studies must disclose on these points even when the disclosure is negative.

Sample size	We used one wild-type zebrafish specimen at 30 days post fertilization. No sample-size calculation was performed. The purpose of the demonstrated imaging experiments was to show the effect of noise suppression and improved imaging contrast through a common biological specimen. Because these effects were systematically tested in a sequence of controlled experiments using ZnO scattering layers, imaging demonstration of one zebrafish is sufficient.
Data exclusions	No data were excluded from the analyses.
Replication	We performed imaging of the 30-dpf zebrafish 3 times and all attempts at replication were successful.
Randomization	This is not relevant to our study as we used a healthy, wild-type zebrafish for the imaging experiments.
Blinding	This is not relevant to our study.

Reporting for specific materials, systems and methods

We require information from authors about some types of materials, experimental systems and methods used in many studies. Here, indicate whether each material, system or method listed is relevant to your study. If you are not sure if a list item applies to your research, read the appropriate section before selecting a response.

Materials & experimental systems

n/a	Involved in the study
☒	☐ Antibodies
☒	☐ Eukaryotic cell lines
☒	☐ Palaeontology
☐	☒ Animals and other organisms
☒	☐ Human research participants
☒	☐ Clinical data

Methods

n/a	Involved in the study
☒	☐ ChIP-seq
☒	☐ Flow cytometry
☒	☐ MRI-based neuroimaging

Animals and other organisms

Policy information about studies involving animals; ARRIVE guidelines recommended for reporting animal research

Laboratory animals	We used a wild-type zebrafish at 30 days after fertilization.
Wild animals	This study did not involve wild animals.
Field-collected samples	This study did not involve samples collected from the field.
Ethics oversight	The fixed zebrafish was provided from Korea University Ansan Hospital. No ethical approval was required for the fixed specimen based on the guidelines of Korea Unversity Institutional Animal Care and Use Committee.

Note that full information on the approval of the study protocol must also be provided in the manuscript.
